# Identification of SPRYD4 as a tumour suppressor predicts prognosis and correlates with immune infiltration in cholangiocarcinoma

**DOI:** 10.1186/s12885-023-10810-9

**Published:** 2023-05-04

**Authors:** Zuyi Ma, Tiange Xie, Jia Sun, Jianchun Yu, Shanzhou Huang, Qi Zhou, Binglu Li

**Affiliations:** 1 Department of General Surgery, State Key Laboratory of Complex Severe and Rare Diseases, Peking Union Medical College Hospital, Chinese Academy of Medical Science and Peking Union Medical College, No. 1 Shuaifuyuan, Dongcheng District, Beijing, 100005 China; 2 Department of General Surgery, Guangdong Provincial People’s Hospital, Guangdong Academy of Medical Sciences, Guangzhou, 510080 China; 3 Department of General Surgery, Hui Ya Hospital of The First Affiliated Hospital, Sun Yat-Sen University, Huizhou, 516081 China; 4 Department of Liver Surgery, The First Affiliated Hospital of Sun Yat-Sen University, Guangzhou, 510000 China

**Keywords:** SPRYD4, Cholangiocarcinoma, Tumor suppressor, Prognosis, Tumor immune infiltration

## Abstract

Cholangiocarcinoma (CCA) is an aggressive solid tumour with a 5-year survival rate ranging from 7% to 20%. It is, therefore, urgent to identify novel biomarkers and therapeutic targets to improve the outcomes of patients with CCA. SPRY-domain containing protein 4 (SPRYD4) contains SPRY domains that modulate protein–protein interaction in various biological processes; however, its role in cancer development is insufficiently explored. This study is the first to identify that SPRYD4 is downregulated in CCA tissues using multiple public datasets and a CCA cohort. Furthermore, the low expression of SPRYD4 was significantly associated with unfavourable clinicopathological characteristics and poor prognosis in patients with CCA, indicating that SPRYD4 could be a prognosis indicator of CCA. In vitro experiments revealed that SPRYD4 overexpression inhibited CCA cells proliferation and migration, whereas the proliferative and migratory capacity of CCA cells was enhanced after SPRYD4 deletion. Moreover, flow cytometry showed that SPRYD4 overexpression triggered the S/G2 cell phase arrest and promoted apoptosis in CCA cells. Furthermore, the tumour-inhibitory effect of SPRYD4 was validated in vivo using xenograft mouse models. SPRYD4 also showed a close association with tumour-infiltrating lymphocytes and important immune checkpoints including PD1, PD-L1 and CTLA4 in CCA. In conclusion, this study elucidated the role of SPRYD4 during CCA development and highlighted SPRYD4 as a novel biomarker and tumour suppressor in CCA.

**Supplementary Information:**

The online version contains supplementary material available at 10.1186/s12885-023-10810-9.

## Introduction

Cholangiocarcinoma (CCA) comprises a heterogeneous group of malignant tumours that are divided into intrahepatic, perihilar and distal CAA depending on the anatomical location. It accounts for more than 15% of all primary hepatobiliary malignancies. The incidence of CAA has gradually increased, with an average growth of 4.4% in the past 10 years [1]. With a 5-year survival rate ranging from 7% to 20%, CCA remains one of the most malignant tumours and accounts for approximately 2% of all cancer-related death worldwide [2]. Curative resection is the only potential treatment option for patients with CCA who were diagnosed at an early stage, whereas, for those diagnosed at an advanced stage, the treatment options are limited. Several clinical trials have proved the clinical benefit of chemotherapy (cisplatin and gemcitabine) as the first-line treatment for unresectable CCA [2, 3]. Additional studies have also identified novel targeted therapies, such as pemigatinib, infigratinib and ivosidenib that have been FDA-approved as second-line treatment options [4, 5]. After decades of research on immune checkpoints inhibitors (ICIs), the use of immunotherapies targeting programmed death-1 (PD-1), its ligand PD-L1 and cytotoxic T lymphocyte antigen 4 (CTLA4) have been reported to be effective in many cancer types. For example, bevacizumab combined with atezolizumab has become the first-line treatment regimen for advanced hepatocellular carcinoma (HCC) [6]. Similar studies on immunotherapies for biliary tract carcinoma (BTC) have attracted much attention [7]. Despite enormous advances in the diagnosis and therapies of cancers, long-term outcomes of patients with CCA remain unsatisfactory owing to its aggressive nature, early tumour recurrence and metastasis. Therefore, it is vital to identify effective biomarkers for early diagnoses and explore targets of driver pathways to better understand the molecular mechanisms involved in CCA development and progression.

Initially identified as the sequence with repeating non-receptor tyrosine kinase spore lysis A (SplA) and ryanodine receptor (RyR), the SPRY domain is one of the most abundant protein domains in mammals [8, 9]. As annotated in SMART [10], several hundred SPRY domains have been identified and classified and approximately 95 SPRY domains have been coded in the human genome [11]. Moreover, accumulating evidence has indicated that the SPRY domains act as protein–protein interaction modules in several biological processes [12]. Specific SPRY domain-containing genes have been reported to play important roles in several human diseases like Opitz syndrome and certain malignancies including melanoma and oral squamous cell carcinoma [8, 13, 14]. Mutations in SPRY domain-containing genes have also been detected in medullary thyroid cancer and endometrial cancer [15, 16]. Notably, SPRY-domain containing protein 4 (SPRYD4), a protein encoded by the SPRYD4 gene, has been demonstrated to serve as a tumour suppressor in HCC and inhibit tumour cell growth by inducing apoptosis [17]. However, its role in CCA remains unexplored.

In this study, the significant downregulation of SPRYD4 was strongly associated with adverse clinical features and outcomes in patients with CCA. Moreover, a strong correlation between SPRYD4 expression and tumour immune infiltration was also demonstrated. Additionally, the overexpression of SPRYD4 inhibited the tumorigenicity and progression of CCA, which was associated with the S/G2 cell phase arrest and enhanced apoptosis. These findings highlighted the role of SPRYD4 as a tumour suppressor, prognostic marker and therapeutic target in CCA.

## Methods

### Data acquisition

RNA-sequencing data of CCA samples were downloaded from The Cancer Genome Atlas (TCGA) database in October 2022. GSE26566 (104 CCA and six bile duct samples), GSE32225 (149 CCA and six bile duct samples), GSE32958 (16 CCA and seven bile duct samples) and GSE76311 (92 CCA and 93 bile duct samples) datasets were obtained from the Gene Expression Omnibus (GEO) database to validate the expression levels of SPRYD4 in CCA. Additionally, the Gene Expression Profiling Interactive Analysis platform (GEPIA) was used to determine SPRYD4 expression levels [18].

### Tissue samples and patient follow-up

We obtained CCA and adjacent bile duct tissue samples from 82 patients with CAA at the Guangdong Provincial People’s Hospital (GPPH cohort) for validation. All enrolled patients were primarily diagnosed with CCA radiographically and confirmed pathologically. Overall survival (OS) was determined as the time from surgical resection to death or last contact, while disease-free survival (DFS) was defined as the period from surgical resection to tumour recurrence or metastasis. All patients were followed up until October 2022, and the median follow-up time was 23.3 months (range, 11.3–51). Enrolled patients’ clinicopathological characteristics including gender, age, carbohydrate antigen 19–9 (CA19-9) index, lymph node metastasis, lymphovascular invasion, tumour differentiation and TNM stage (AJCC 8th edition) were collated for correlation analysis. Additionally, the independent risk factors were identified using univariate and multivariate cox regression analyses. To evaluate the predictive value, Kaplan–Meier analyses were performed in the high-SPRYD4 and low-SPRYD4 groups in the GPPH cohort. All enrolled patients provided their written informed consent before participation. The study was approved by the Ethics Association of Guangdong Provincial People’s Hospital.

### Functional enrichment analyses

The genes that co-expressed with SPRYD4 (|Spearman’s correlation coefficient|> 0.45 and *P* < 0.05) were screened using cBioPortal. The Gene Ontology (GO), Kyoto Encyclopaedia of Genes and Genomes (KEGG) and Gene set enrichment analysis (GSEA) analyses were performed as described previously [19]. The results were visualised using the R package ‘ggplot2’. To analyse the associations between SPRYD4 and its neighbouring genes, GeneMANIA and STRING databases were used to establish a gene network map and protein–protein interaction (PPI) network [20, 21].

### Multiple immune infiltration analyses

Based on an integrated web portal TISIDB for tumour and immune system interaction analysis [22], we evaluated the relationships between SPRYD4 expression and immunostimulators, immunoinhibitors, tumour infiltrating lymphocytes including CD8^+^ T cells, CD4^+^ T cells, B cells, macrophages, dendritic cells, natural killer cells and neutrophils in CCA. Additionally, the correlations between SPRYD4 expression and gene markers of immune-infiltrating cells and immune checkpoints were analysed using Spearman’s correlation coefficients.

### Cell culture and transfection

We obtained CCA cell strains including HUCCT1, RBE, CCLP-1, huh28, QBC939 and HCCC-9810 from Procell (Wuhan, China). All cells were cultured in Roswell Park Memorial Institute 1640 medium (Gibco, USA) supplemented with 10% foetal bovine serum and incubated at 37℃ with 5% CO_2_. An EGFP-Puro-CMV-3 × Flag hSPRYD4 vector was constructed by Generay Biotech Co., Ltd. (Shanghai, China). Mixed with the pPACKH1 packaging plasmid, the SPRYD4-OV vector was transfected into 293 T cells. The virus particles were collected from the concentrated virus precipitation solution following the SBI instructions. The cells were infected with TUNDUX virus transducers. Following this, puromycin screening was used to identify the positive cells. For the generation of cell lines with SPRYD4 knockdown, two siRNAs targeting SPRYD4 were transfected into CCA cells to silence SPRYD4. The siRNAs used in the study were obtained from sequences (5’- 3’) shown below:

1. GCCCCAAAUCAGAAAAUAAAC
2. AAGGCGCAAGAUGGCGCUGCU

### Cell proliferation and migration assays

Cell proliferation and migration assays were conducted as previously described [23].

### Flow cytometry

CCA cells were harvested, fixed in 75% ethanol and stored at 4℃ overnight. Then, the cells were pre-treated and stained with Propidium (PI) & RNase Staining Buffer (SIGMA) for cell cycle analysis, while Annexin V-APC (Invitrogen) was used for apoptosis detection. The percentage of CCA cells at different stages and statuses were evaluated using flow cytometry (Novocyte D2060R, Agilent).

### Real-time reverse transcription quantitative polymerase chain reaction (RT-qPCR) and immunohistochemistry (IHC)

RT-qPCR and IHC were performed as described previously [23]. The primer pairs used for RT-qPCR are listed in Table S1. TUNEL Detection Kit (Alexa Fluor 640) was obtained from Yeasen Biotechnology (Shanghai, China).

### Animal experiments

To establish xenograft mouse models, 1 × 10^6^ HUCCT1 SPRYD4-OV and control group cells were injected subcutaneously into 6-week-old male BALB/c-nude mice (GemPharmatech, China) (*n* = 6 per group). The tumour volume was measured and recorded using a calliper every 10 days. Fifty days after implantation, the mice were sacrificed by dislocation of cervical vertebra and then the xenografts were removed, weighed, recorded and used in IHC analysis to detect the levels of SPRYD4, Ki67 and Tunel. The Ethics Association of Guangdong Provincial People’s Hospital approved the animal experiments. All 6-week old male BALB/c nude mice were purchased from GemPharmatech (Jiangsu, China).

### Statistical analysis

Student’s t-test was performed for the statistical analysis between continuous parameters, while χ2 test or Fisher’s exact test was used to evaluate qualitative variables in vivo and in vitro. All statistical analyses were performed using the SPSS version 24.0 and R software version 4.0.1. *P* < 0.05 was considered statistically significant.

## Results

### Low SPRYD4 expression in CCA

**Fig. 1:**
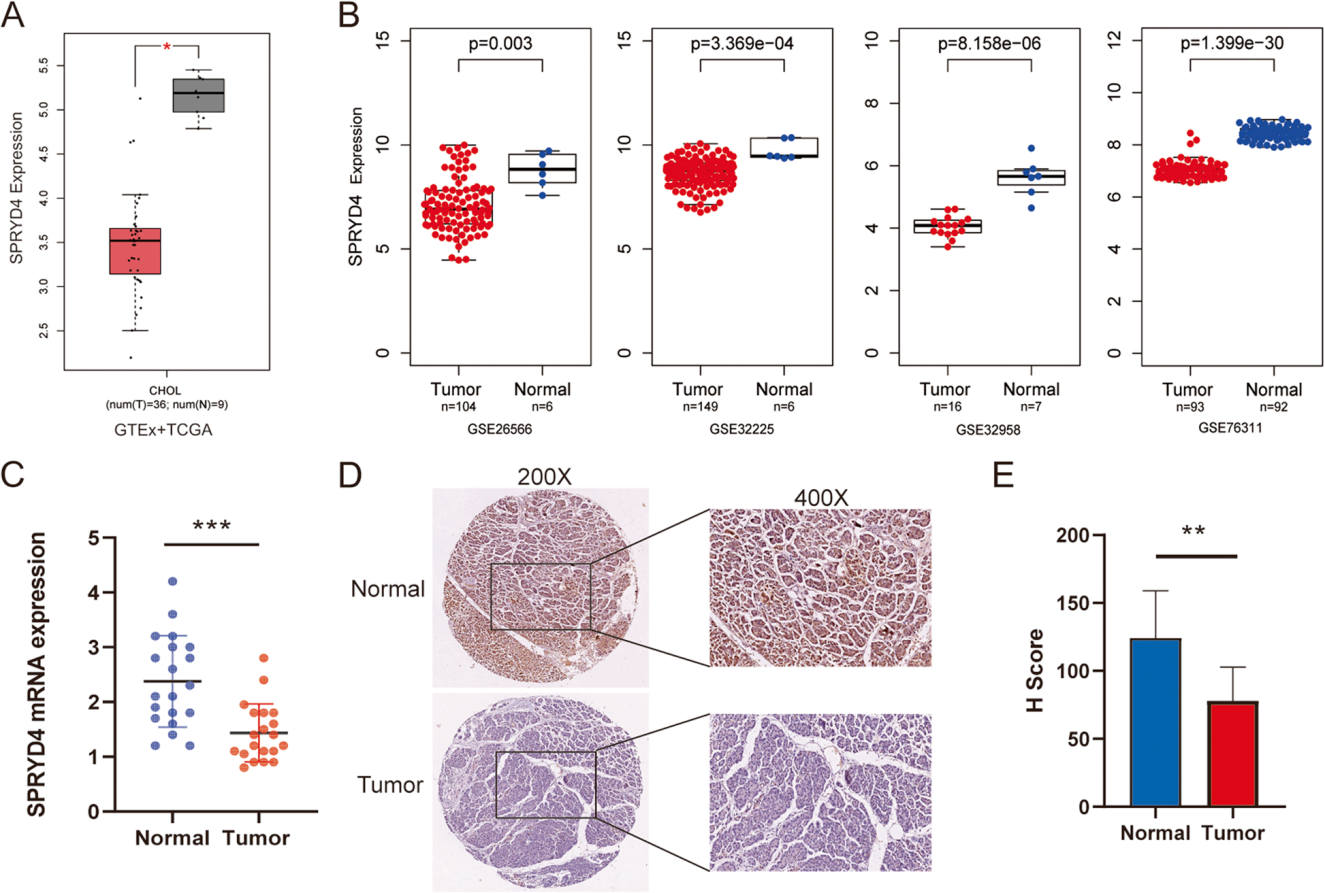
SPRYD4 is low-expressed in CCA. **A** Low-expression of SPRYD4 in CCA was identified in GEPIA database. **B** Down-regulation of SPRYD4 was identified in CCA in four individual GEO datasets (GSE26566, GSE32225, GSE32958 and GSE76311). **C** The mRNA over-expression levels of SPRYD4 were identified in CCA tissues and normal bile duct tissues of 20 samples. **D** Representative images of SPRYD4 staining in CCA specimens and normal bile duct tissues. **E** Immunohistochemistry staining showed the protein levels of SPRYD4 were down-regulated in CCA tissues. Scale bars: 200 μm. *P*-values were determined by Non-parametric Mann–Whitney U-test in A-B. *P*-values were assessed by two-tailed t-tests in C and E. All * *P*-value < 0.05, ** *P*-value < 0.01, *** *P*-value < 0.001

Bioinformatics analyses were performed to evaluate the differential expression of SPRYD4 in several cancer types. The GEPIA database suggested that SPRYD4 expression was downregulated in some cancer types especially CCA (Figs. 1A and S1). Four datasets acquired from the GEO platform, namely GSE26566, GSE32225, GSE32958 and GSE76311, were used to identify the low-expressing samples of SPRYD4 in CCA compared to normal bile duct tissues (Fig. 1B). Furthermore, the RT-qPCR assay of 20 fresh CCA and normal bile duct tissues revealed that the mRNA levels of SPRYD4 were downregulated in the tumour tissues (Fig. 1C). Moreover, the low levels of SPRYD4 protein in CCA tissues were validated by IHC (Fig. 1D-E).

### Loss of SPRYD4 correlates with unfavourable clinicopathological characteristics and poor prognosis in CCA

**Fig. 2:**
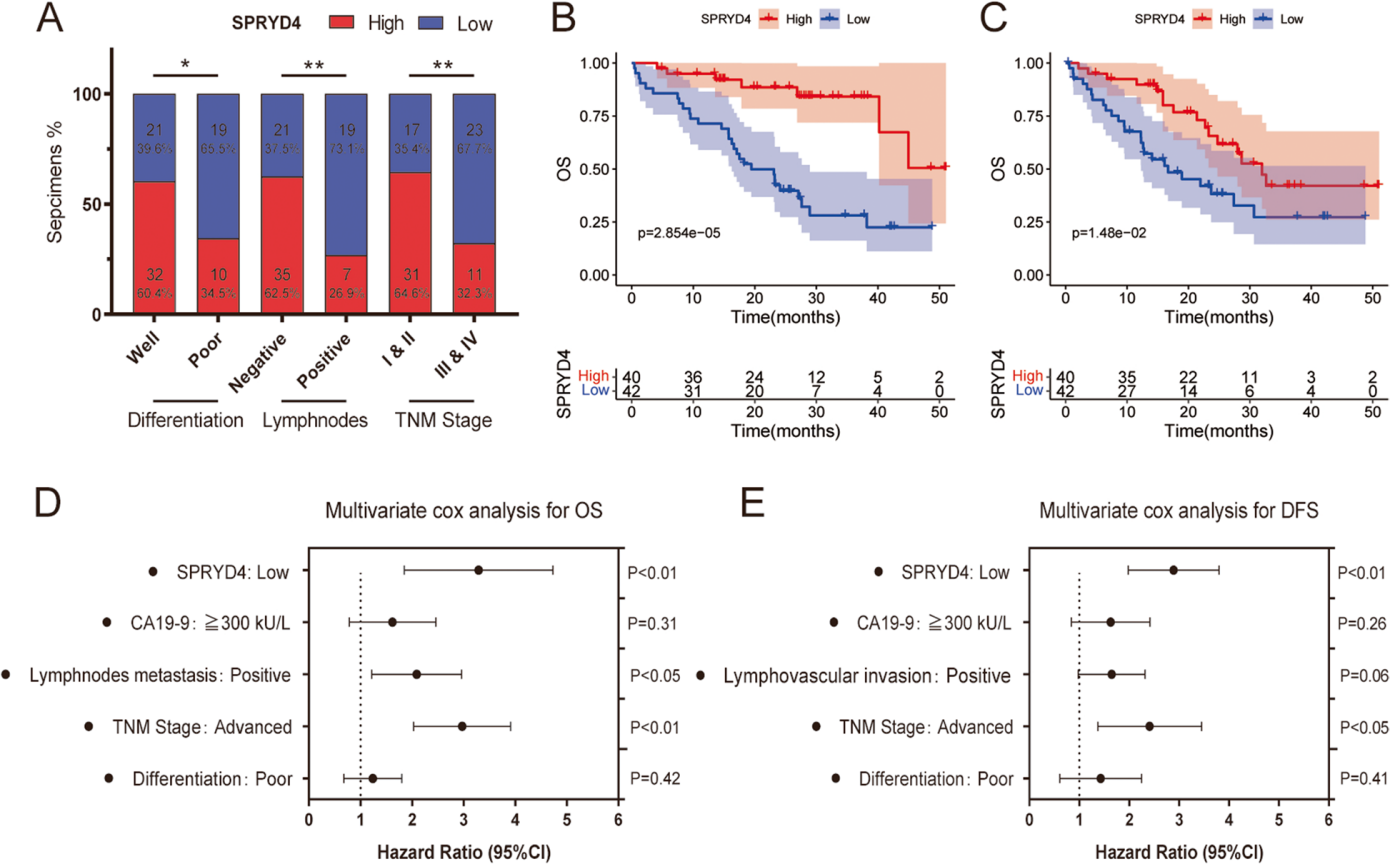
Loss of SPRYD4 correlates with unfavorable clinicopathological characteristics and poor prognosis in CCA. **A** SPRYD4 expression of CCA was significantly correlated with tumor differentiation, lymphnode metastasis and TNM stage. **B-C** Kaplan–Meier analyses showed CCA patients with low expression of SPRYD4 had inferior OS (**B**) and DFS (**C**) than those with high expression in GPPH cohort. **D-E** Multivariate Cox regression analyses were performed to confirmed low expression of SPRYD4 was independent risk factor for OS (**D**) and DFS (**E**) of CCA patients from GPPH cohort. *P*-values were determined by χ2 tests or Fisher’s exact tests in **A**. The Hazard Ratios (HR) and* P*-values by the log-rank (Mantel-Cox) test are calculated in **B**-**C**. All * *P*-value < 0.05, ** *P*-value < 0.01

A total of 82 patients with CCA were classified into high-SPRYD4 and low-SPRYD4 groups based on IHC analysis. As shown in Fig. 2A and Table 1, the downregulation of SPRYD4 in CCA was significantly correlated with poor tumour differentiation, positive lymph node metastasis and advanced TNM stage. Moreover, Kaplan–Meier analyses of the GPPH cohort suggested that patients with CCA exhibiting low SPRYD4 expression had poorer OS (*P* = 2.854e-05) and DFS (*P* = 1.48e-02) than those with high expression (Fig. 2B-C). Univariate and multivariate Cox regression analyses also suggested that low SPRYD4 expression was the independent prognostic risk factor for OS and DFS of patients with CCA (Fig. 2D-E and Tables 2 and 3). Additionally, patients with low SPRYD4 expression also had poor OS in other cancer types including kidney renal clear cell carcinoma (KIRC), liver hepatocellular carcinoma (LIHC), pancreatic adenocarcinoma (PAAD) and lung squamous cell carcinoma (LUSC) (Fig. S2). Thus, these findings indicate that SPRYD4 can serve as a potential prognosis indicator of CCA.

**Table 1:** Correlation between SPRYD4 expression with clinicopathological characteristics of ACC patients

Clinicopathological variables	Patients (*n* = 82)	SPRYD4 expression		*P* Value
		High (42)	Low (40)	
Gender				
Male	52	25	27	0.60
Female	30	17	13	
Age				
≧50	50	28	22	0.39
< 50	32	14	18	
CA19-9				
≧300 kU/L	31	13	18	0.28
< 300 kU/L	51	29	22	
Lymphnodes metastasis				
Positive	26	7	19	**< 0.01**
Negative	56	35	21	
Lymphovascular invasion				
Positive	18	8	10	0.70
Negative	64	34	30	
TNM stage				
Advanced (III & IV)	34	11	23	**< 0.01**
Early (I & II)	48	31	17	
Differentiation				
Poor	29	10	19	**< 0.05**
Well	53	32	21

**Table 2:** Univariate and multivariate Cox regression analysis of risk factors associated with overall survival

Clinicopathological variables	Univariate analysis			Multivariate analysis		
	HR	95% CI	*P* Value	HR	95% CI	*P* Value
SPRYD4 expression (Low vs. High)	6.81	5.23–8.39	**< 0.01**	3.29	1.85–4.73	**< 0.01**
Gender (Male vs. Female)	1.72	0.83–2.61	0.25			
Age (≧50 vs. < 50)	1.31	0.34–2.28	0.67			
CA19-9 (≧300 kU/L vs. < 300 kU/L)	2.09	1.14–3.04	**< 0.05**	1.62	0.78–2.46	0.31
Lymphnodes metastasis (Positive vs. Negative)	2.96	1.99–3.93	**< 0.01**	2.09	1.22–2.96	**< 0.05**
Lymphovascular invasion (Positive vs. Negative)	1.51	0.42–2.60	0.58			
TNM stage (Advanced vs. Early)	4.57	3.34–5.8	**< 0.01**	2.97	2.03–3.91	**< 0.01**
Differentiation (Poor vs. Well)	2.04	1.11–2.97	**< 0.05**	1.24	0.56–1.92	0.42

**Table 3:** Univariate and multivariate Cox regression analysis of risk factors associated with disease-free survival

Clinicopathological variables	Univariate analysis			Multivariate analysis		
	HR	95% CI	*P* Value	HR	95% CI	*P* Value
SPRYD4 expression (Low vs. High)	5.83	4.16–7.50	**< 0.01**	2.89	1.98–3.80	**< 0.01**
Gender (Male vs. Female)	1.32	0.29–2.35	0.75			
Age (≧50 vs. < 50)	1.26	0.41–2.11	0.83			
CA19-9 (≧300 kU/L vs. < 300 kU/L)	2.34	1.26–3.42	**< 0.05**	1.63	0.84–2.42	0.26
Lymphnodes metastasis (Positive vs. Negative)	1.51	0.62–2.40	0.43			
Lymphovascular invasion (Positive vs. Negative)	2.97	2.15–3.79	**< 0.01**	1.65	0.98–2.82	0.06
TNM stage (Advanced vs. Early)	4.09	2.89–5.29	**< 0.01**	2.41	1.37–3.45	**< 0.05**
Differentiation (Poor vs. Well)	2.07	1.19–2.95	**< 0.05**	1.43	0.61–2.12	0.41

### Neighbour gene network of SPRYD4 and functional enrichment analyses in CCA

**Fig. 3:**
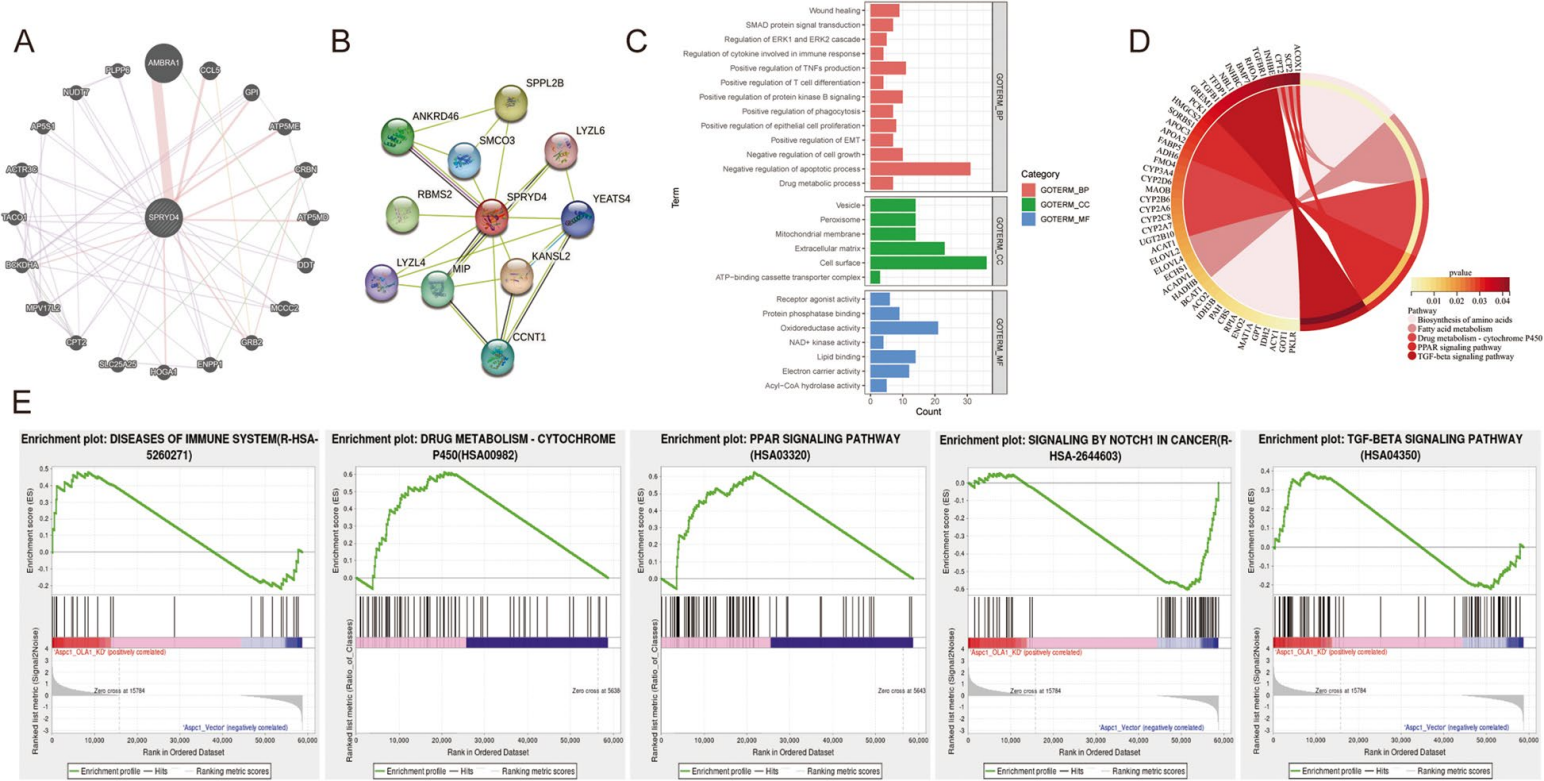
Neighbor gene network of SPRYD4 and functional enrichment analyses in CCA. **A** GeneMANIA tool was used to show the relationships of neighbor genes of SPRYD4 and construct a network map. **B** STRING tool was used to construct a protein–protein interaction network (PPI) of SPRYD4. **C** GO enrichment analysis of SPRYD4. **D** KEGG pathway analysis of SPRYD4. **E** GSEA of SPRYD4

The relationships between SPRYD4 with its neighbour genes were evaluated and a corresponding network was constructed using GeneMANIA and STRING. As shown in the map, the central node (SPRYD4) was surrounded by 20 nodes, which represented closely related genes, namely *AMBRA1, CCL5, GPI, ATP5ME, CRBN, ATP5MD, DDT, MCCC2, GRB2, ENPP1, HOGA1, SLC25A25, CPT2, MPV17L2, BCKDHA, TACO1, ACTR3C, AP5S1, NUDT7* and *PLPP6*. Furthermore, the PPI network also indicated that SPRYD4 correlates with *SPPL2B, LYZL6, YEATS4, KANSL2, CCNT1, LYZL4, RBMS2, ANKRD46* and *SMCO3* (Fig. 3A-B).

To further explore the potential function of SPRYD4, 719 co-expressed genes of SPRYD4 were screened using cBioPortal and subjected to DAVID 6.8 for GO and KEGG analyses. Together with the co-expressed genes, GO analysis revealed that SPRYD4 regulated certain biological processes including cell growth, epithelial cell proliferation, phagocytosis, apoptosis, epithelial–mesenchymal transition, ERK1 and ERK2 cascade, tumor necrosis factors production, T cell differentiation and cytokine regulation in immune response and drug metabolic process (Fig. 3C). Furthermore, KEGG analysis revealed that SPRYD4 was involved in drug metabolism-cytochrome P450, the biosynthesis of amino acids, the PPAR signalling pathway, fatty acid metabolism and the TGF-β signalling pathway (Fig. 3D). Additionally, GSEA was used to explore differentially enriched pathways between high- and low-SPRYD4 expression groups. The significantly enriched biological processes included diseases of immune system, drug metabolism-cytochrome P450, PPAR signalling pathway, NOTCH1 signalling in cancer and the TGF-βsignalling pathway (Fig. 3E).

### SPRYD4 participates in CCA cell proliferation and migration

**Fig. 4:**
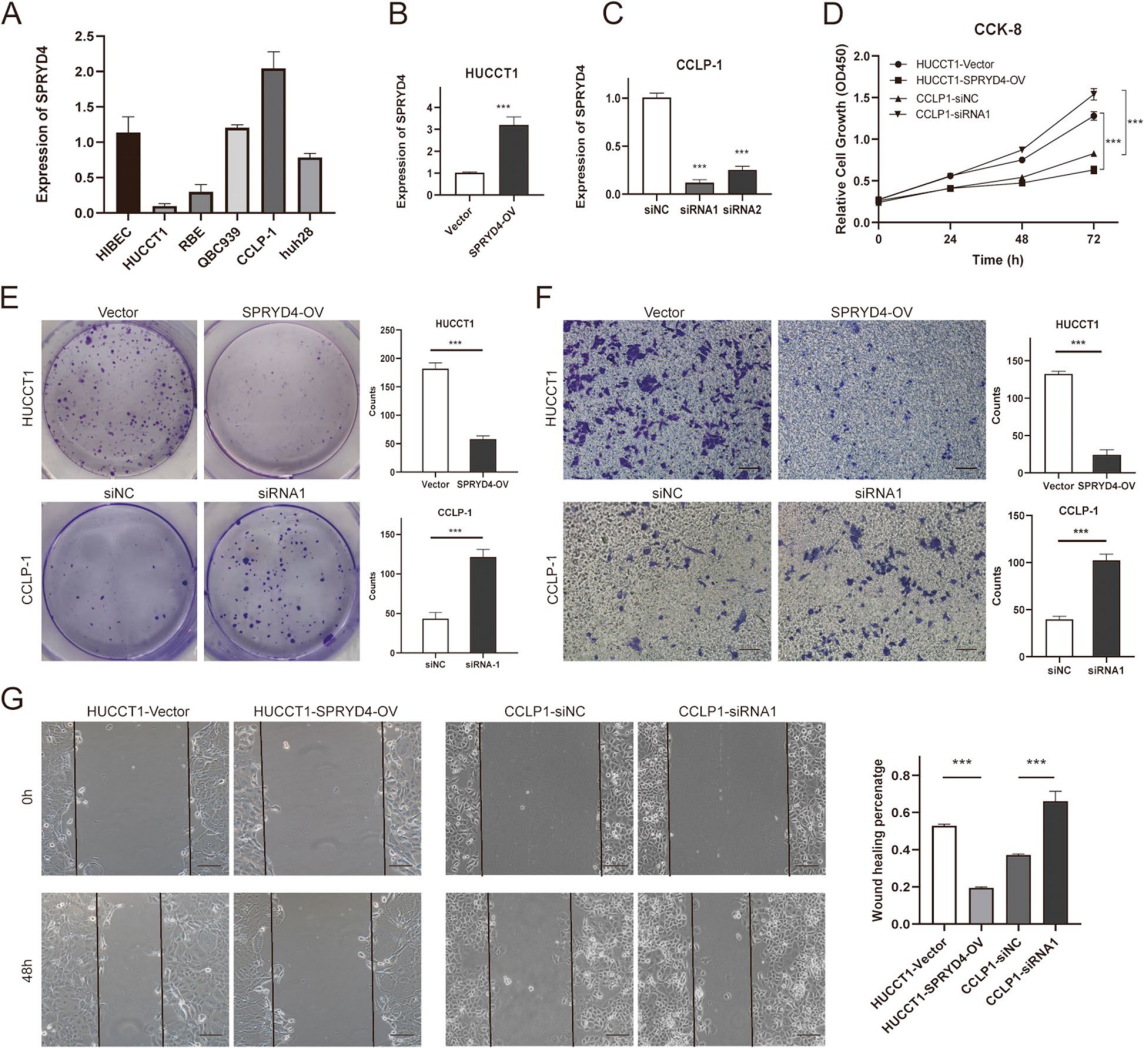
SPRYD4 participates in CCA cells proliferation and migration. **A** Rt-qPCR showed SPRYD4 was highly expressed in CCLP-1 cells, while relatively low-expressed in HUCCT1 cells among CCA cell lines. **B-C** RT-qPCR were used to detect the over-expression of SPRYD4 in HUCCT1 cells (**B**) and the knock-down of SPRYD4 in CCLP-1 cells (**C**) after transfection. **D-E** CCK-8 assay (**D**) and colony formation assay (**E**) showed that SPRYD4 over-expression reduced the proliferation of HUCCT1 cells, while SPRYD4 knock-down promoted the proliferation of CCLP-1 cells. **F-G** Transwell assay (**F**) and wound-healing assay (**G**) showed that SPRYD4 over-expression reduced the migration of HUCCT1 cells, while SPRYD4 knock-down promoted the migration of CCLP-1 cells. Scale bars: 100 μm. *P*-values were assessed using two-tailed t-tests and ANOVA followed by Dunnett’s tests for multiple comparison in **B**-**G**. All *** *P*-value < 0.001

The expression levels of SPRYD4 were determined in several CCA cell strains. Low SPRYD4 expression was observed in HUCTT1 cells whereas high SPRYD4 expression was observed in the CCLP-1 cell line (Fig. 4A). The HUCTT1 cell line was established with a stable SPRYD4 over-expression (HUCTT1/SPRYD4-OV) for gain-of-function analysis (Fig. 4B), while two si-SPRYD4 were transfected into CCLP-1 cells to obtain CCLP-1/SPRYD4-KD for loss-of-function analysis (Fig. 4C). Following the validation of SPRYD4 expression using RT-qPCR, CCK-8 and colony formation analyses were performed, wherein the over-expression of SPRYD4 suppressed the proliferative ability of HUCTT1 cells, while the silencing of SPRYD4 augmented CCLP-1 cell proliferation (Fig. 4D-E). Additionally, transwell and wound healing assays demonstrated that migration was inhibited in SPRYD4-OV cells. Conversely, the downregulation of SPRYD4 promoted the migratory activity in SPRYD4-KD cells (Fig. 4F-G). These findings indicate that the over-expression of SPRYD4 inhibits CCA cell proliferation and migration.

### The association between SPRYD4 expression and immune infiltration in CCA

**Fig. 5:**
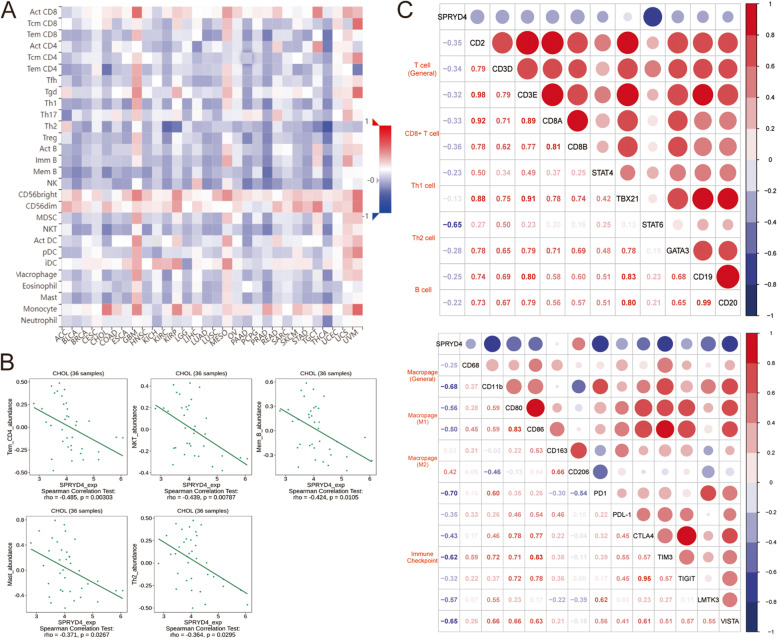
The association between SPRYD4 expression and immune infiltration in CCA. **A** Correlations between SPRYD4 expression and TILs shown by TISIDB web server. **B** Top 5 TILs with greatest negative Spearman’s correlation with SPRYD4. **C** Correlations between SPRYD4 expression and gene markers from TILs and other immune checkpoints. Spearman’s correlation was performed in **B** and **C**

Tumour infiltrating lymphocytes (TILs), such as T cells, B cells, macrophages and NK cells, play an essential role in cancer development and mediating chemotherapy response in several cancer types, including CAA [24]. Functional enrichment analysis indicated that SPRYD4 may be involved in immune reactions; therefore, we explored the association between SPRYD4 expression and tumour immune infiltration in CCA. In the TISIDB database, SPRYD4 showed a negative correlation with TILs in CCA (Fig. 5A). SPRYD4 was negatively correlated with the presence of effector memory CD4^+^ T cells (*R* = -0.485, *P* = 0.00303), NK cells (*R* = -0.439, *P* = 0.00787), memory B cells (*R* = -0.424, *P* = 0.0105), mast cells (*R* = -0.371, *P* = 0.0267) and type 2 helper T cells (*R* = -0.364, *P* = 0.0295) (Fig. 5B). Additionally, Fig. 5C displays the negative correlations between SPRYD4 expression and general T cells (CD2, CD3D and CD3E), CD8^+^ T cells (CD8A and CD8B), Th2 cells (STAT6), macrophages (CD68, CD11b, CD80 and CD86) and immune checkpoints including PD1, PD-L1, CTLA4, TIM3, TIGIT, LMTK3 and VISTA.

To further investigate the role of SPRYD4 in tumour immune infiltration, we evaluated the associations between SPRYD4 expression and immunostimulators or immunoinhibitors (Fig. S3A-B). Regarding immunostimulators, SPRYD4 was negatively associated with TNFSF4 (*R* = -0.483, *P* = 0.00314), PVR (*R* = -0.461, *P* = 0.00509) and ENTPD1 (*R* = -0.462, *P* = 0.00509) (Fig S3C). However, in terms of immunoinhibitors, SPRYD4 was negatively associated with TGFBR1 (*R* = -0.471, *P* = 0.00412), TGFB1 (*R* = -0.473, *P* = 0.00397) and CTLA4 (*R* = -0.321, *P* = 0.0564) (Fig. S3D). Thus, these findings reveal that SPRYD4 has the potential to mediate tumour-specific immune responses by regulating TILs and immune-related molecules.

### SPRYD4 plays a key role in the cell cycle and apoptosis in CCA cells

**Fig. 6:**
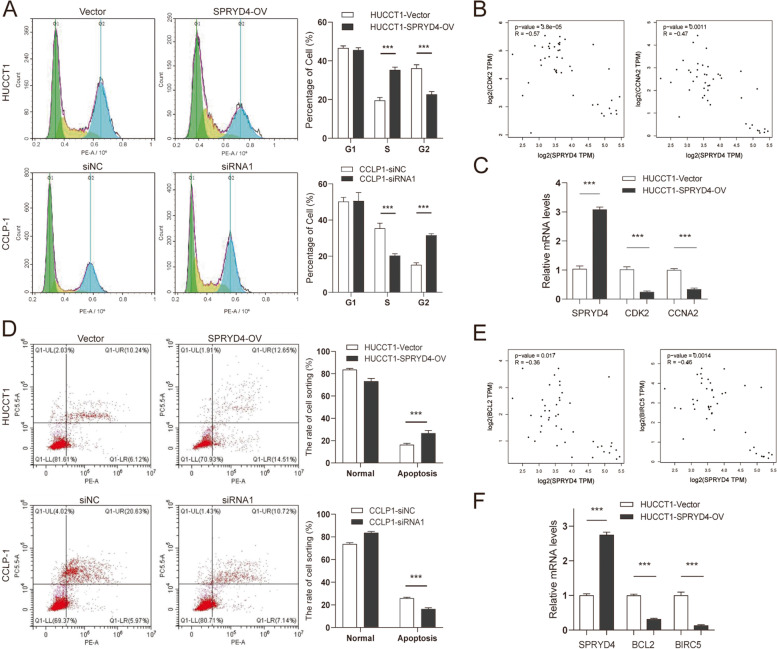
SPRYD4 plays a key role in cell cycle and apoptosis in CCA cells. **A** Flow cytometry showed over-expression of SPRYD4 triggered G2/S blockade in HUCCT1 cells, while depletion of SPRYD4 increased the percentage of S phase in CCLP-1 cells. **B** Correlations of SPRYD4 expression and CDK2, CCNA2 shown by GEPIA web server. **C** RT-qPCR validated the expression of CDK2 and CCNA2 was down-regulated in HUCCT1/SPRYD4-OV cells. **D** Flow cytometry showed that SPRYD4 over-expression increased the percentage of apoptotic HUCCT1 cells, while SPRYD4 deletion reduced the percentage of apoptotic CCLP-1 cells. **E** Correlations of SPRYD4 expression and BCL-2, BIRC5 shown by GEPIA web server. **F** RT-qPCR validated the expression of BCL-2 and BIRC5 (Survivin) was down-regulated in CCLP1/SPRYD4-KD cells. *P*-values were assessed using two-tailed t-tests in **A**, **C**, **D** and **F**. Spearman’s correlation was performed in **B** and **E**. All *** *P*-value < 0.001

To further validate the enrichment of SPRYD4 in cell growth and apoptosis, which was revealed using GO analysis, we performed flow cytometry to explore the specific function of SPRYD4 in the CCA cell cycle and apoptosis. As indicated in Fig. 6A, the over-expression of SPRYD4 inhibited the cell cycle of HUCTT1 cells at the S/G2 stage, while the CCLP-1/SPRYD4-KD cell line exhibited an increased percentage of cells in the S phase. GEPIA analysis further indicated that SPRYD4 was negatively correlated with CDK2 (*R* = -0.57, *P* = 3.8e-05) and CCNA2 (*R* = -0.47, *P* = 0.0011) (Fig. 6B). Following this, the low expressions of CDK2 and CCNA2 were validated in SPRYD4-OV cells using RT-qPCR (Fig. 6C). The over-expression of SPRYD4 increased the proportion of apoptotic HUCTT1 cells, while the knockdown of SPRYD4 decreased the percentage of apoptotic CCLP-1 cells (Fig. 6D). Furthermore, negative correlations were also observed between SPRYD4 and BCL-2, BIRC5 in GEPIA (Fig. 6E). Additionally, the upregulation of SPRYD4 reduced the mRNA level of BCL-2 and BIRC5/Survivin in CCA cells (Fig. 6F). Taken together, SPRYD4 arrests S/G2 progression and activates the apoptosis pathway in CCA cells.

### SPRYD4 over-expression inhibits tumour growth in vivo

**Fig. 7:**
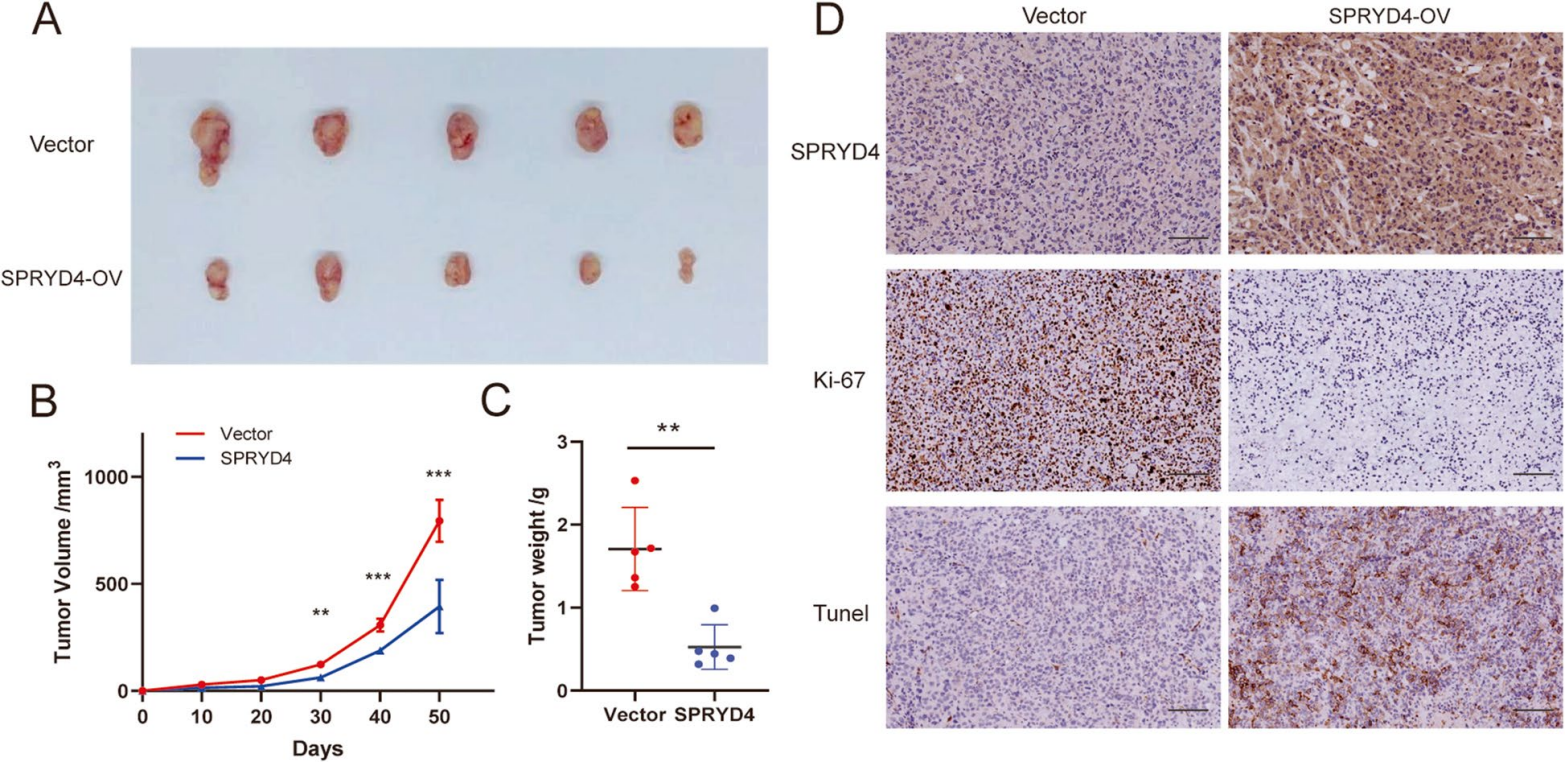
SPRYD4 over-expression inhibits tumor growth in vivo. **A** BALB/c-nudes (*n* = 6 per group) were sacrificed 50 days after the injection and tumors dissected from respective groups were shown. **B** Tumor growth curves after the injection of HUCCT1/SPRYD4-OV and control cells. Tumor volume was calculated every 10 days. **C** Tumor weight was measured in SPRYD4-OV and control groups. **D** IHC staining of SPRYD4, Ki67 and Tunel in tumors from SPRYD4-OV and control groups. Scale bars: 200 μm. *P*-values were assessed using two-tailed t-tests and ANOVA followed by Dunnett’s tests for multiple comparison in **B**-**C**. All ** *P*-value < 0.01, *** *P*-value < 0.001

HUCCT1 SPRYD4-OV and control cells were injected in BALB/c-nude mice and a xenograft mouse model was constructed to assess the influences of SPRYD4 on tumour growth in vivo. The over-expression of SPRYD4 significantly suppressed tumour growth rate 50 days after injection (Fig. 7A-B). Moreover, a significant decrease in tumour weight in the SPRYD4-OV group was observed at the end of the experiment compared to the control group (Fig. 7C). Additionally, IHC assays indicated a high expression of Ki-67 in SPRYD4-OV tumour tissues, while a high expression of Tunel was observed in the control group (Fig. 7D). Collectively, the in vivo experiments indicated that the over-expression of SPRYD4 inhibited CCA tumour growth in vivo.

## Discussion

Identification of novel biomarkers of CCA development and progression aids in the development of therapeutic targets and improvement of outcomes in patients with CCA. To the best of our knowledge, this study is the first to identify that SPRYD4 was downregulated in CCA tissues compared to normal bile duct tissues and works as a tumour suppressor in CCA. Moreover, low SPRYD4 expression in CCA was also associated with poor tumour differentiation, positive lymph node metastasis and advanced TNM stage. In the study cohort, patients with CCA having low SPRYD4 expression had the worst survival, highlighting the predictive ability of SPRYD4 in CCA prognosis. Furthermore, in vitro experiments revealed that SPRYD4 over-expression inhibited CCA cell proliferation and migration, while the proliferative and migratory capacity of CCA cells was significantly enhanced after SPRYD4 deletion. Additionally, the tumour-inhibitory effect of SPRYD4 was validated in vivo using xenograft mouse models.

The uncontrolled proliferation of tumour cells is the most vital biological behaviour of malignancies, which is mainly based on the dysfunction of the cell cycle and apoptosis regulation [25, 26]. Functional enrichment analyses revealed that SPRYD4 and its neighbour genes were mainly enriched in cell growth, cell proliferation and apoptotic processes, which were further validated using flow cytometry. Consistent with previous reports [17], the over-expression of SPRYD4 triggered the S/G2 phase arrest and promoted apoptosis in CCA cells. Accumulating evidence has suggested that cytokine-dependent kinase 2 (CDK2) plays an essential role in cell cycle transition and DNA repair response (DDR) [27, 28]. Activated by binding to Cyclin A2 (CCNA2) and cyclin E1/2 (CCNE1/2), CDK2 is a vital cell-cycle regulator that drives G1/S transition, which regulated DNA initiation and replication throughout the S phase [29, 30]. In the current study, bioinformatic analyses revealed that the expression of SPRYD4 correlated negatively with CDK2 and CCNA2. Thus, after over-expressing SPRYD4, the expression of CDK2 and CCNA2 was proportionally attenuated in CCA cells, which explained the S-phase arrest in SPRYD4-OV cells. Furthermore, SPRYD4 expression also negatively correlates with two notable apoptotic inhibitors, BCL-2 and BIRC5. Herein, SPRYD4 over-expression significantly inhibited BCL-2 and BIRC5/survivin expression. Reed et al. initially identified BCL-2 as an anti-apoptotic gene that was associated with tumorigenesis and cancer progression [31, 32]. In apoptosis pathways, BCL-2 inhibited cytochrome c that was released from the mitochondria to prevent caspases activation, which was directly responsible for cell apoptosis [33]. Apart from apoptosis, BCL-2 also played essential roles in angiogenesis and chemotherapy resistance [34, 35]. This study revealed that SPRYD4 could regulate BCL-2 levels; however, its specific effect on angiogenesis and chemoresistance requires further study.

The tumour immune microenvironment has been vastly studied in recent years, which resulted in immunotherapy advancements that regulate immune responses against tumour cells [36]. As the most vital determinants of the cancer-related immune response, TILs are responsible for tumour immune surveillance and cancer cell elimination. In our study, GO analysis revealed the involvement of SPRYD4 in immune reactions such as T-cell differentiation and immune response-related cytokine regulation. Notably, SPRYD4 expression was negatively correlated with specific TILs, especially NK T cells, effector memory CD4^+^ T cells and memory B cells in CCA. NKT cells are powerful immune regulators that modulate immune responses by secreting either Th1-, Th2-, Th17- or Treg-cell-associated cytokines [37, 38]. Therefore, NKT cells are also considered essential mediators of cancer immune surveillance. To escape immune surveillance, tumour cells express high levels of immune checkpoints, such as PD-1/PD-L1 and CTLA4, which aid in escaping the tumour-killing effect of TILs [39]. As one of the most indispensable checkpoints, PD-1 binds to PD-L1, leading to T-cell dysfunction and apoptosis [39]. Similarly, CTLA4, an inhibitory receptor that is expressed in T cells, inhibits T-cell activation, highlighting its effectiveness in cancer treatment [40, 41]. To date, ICIs are a hotspot in oncological therapy. Recent clinical trials have demonstrated the significant efficacy of pembrolizumab (PD-1 inhibitor) and ipilimumab (CTLA4 inhibitor) in anti-tumour activity, bringing in a new dawn in CCA treatment [42]. In the present study, SPRYD4 showed a negative correlation with various important immune checkpoint markers including PD1, PD-L1, CTLA4, TIM3, TIGIT, LMTK3 and VISTA in CCA. However, further studies are required to explore the relationship and underlying regulatory mechanism of SPRYD4 expression with immune infiltration and checkpoints in CCA.

## Conclusion

Our study demonstrated that the low expression of SPRYD4 correlated with adverse clinical characteristics and prognosis in patients with CCA. Moreover, in vitro studies suggested that the overexpression of SPRYD4 impaired S/G2 progression in the cell cycle and promoted cell apoptosis, which inhibited CCA initiation and progression. Thus, SPRYD4, a tumour suppressor, can be a potential indicator for CCA prognosis. However, further studies are required to validate its predictive ability.

## Supplementary Information

**Additional file 1: Table S1.** Information on PCR primer oligonucleotide sequences.

**Additional file 2: Figure S1. **SPRYD4 expressions in pan-cancer. The analyses of SPRYD4 expression in several cancer types in GEPIA database. **Figure S2. **Survival analyses of SPRYD4 in other cancer types. Kaplan-Meier analyses showed patients with low SPRYD4 expression had inferior overall survival in kidney renal clear cell carcinoma (KIRC), kidney renal papillary cell carcinoma (KIRC), liver hepatocellular carcinoma (LIHC), pancreatic adenocarcinoma (PAAD) and lung squamous cell carcinoma (LUSC). **Figure S3.** Correlations of SPRYD4 with immunomodulators in ACC. (A) Correlations between immunostimulators and SPRYD4 expression shown by TISIDB database. (B) Correlations between immunoinhibitors and SPRYD4 expression shown by TISIDB database. (C) Top 3 immunostimulators with greatest negative Spearman’s correlation with SPRYD4. (D) Top 3 immunoinhibitors with greatest negative Spearman’s correlation with SPRYD4.

## Data Availability

RNA-sequencing data can be obtained from TCGA (https://portal.gdc.cancer.gov/repository), GEO (https://www.ncbi.nlm.nih.gov/geo/) and GEPIA (http://gepia.cancer-pku.cn/index.html). CBioPortal (https://www.cbioportal.org/), GeneMANIA (http://genemania.org/) and STRING (https://string-db.org/cgi/) databases were used in functional enrichment analyses. TISIDB (http://cis.hku.hk/) was used in immune infiltration analyses. Other data that support the findings of this study are available from the corresponding author upon reasonable request.

## Abbreviations

CCA: Cholangiocarcinoma
ICPIs: Immune checkpoints inhibitors
HCC: Hepatocellular carcinoma
BTC: Biliary tract carcinoma
PD-1: Programmed death-1
PD-L1: Programmed death-ligand 1
SplA: Spore lysis A
RyR: Ryanodine receptor
SMART: Simple Modular Architecture Research Tool
SPRYD4: SPRY-domain containing protein 4
TCGA: The Cancer Genome Atlas
GEO: Gene Expression Omnibus
GEPIA: Gene Expression Profiling Interactive Analysis
OS: Overall survival
DFS: Disease-free survival
CA19-9: Carbohydrate antigen 19–9
GO: Gene Ontology
KEGG: Kyoto Encyclopedia of Genes and Genomes
GSEA: Gene set enrichment analysis
PPI: Protein–protein interaction
RPMI: Roswell Park Memorial Institute
FBS: Fetal bovine serum
RT-qPCR: Real-time reverse transcription quantitative PCR
IHC: Immunohistochemistry
KIRC: Kidney renal clear cell carcinoma
KIRC: Kidney renal papillary cell carcinoma
LIHC: Liver hepatocellular carcinoma
PAAD: Pancreatic adenocarcinoma
LUSC: Lung squamous cell carcinoma
TILs: Tumor infiltrating lymphocytes
CCNA2: Cyclin A2
CCNE1/2: Cyclin E1/2
TIME: Tumor immune microenvironment
NKT cells: Natural killer T cells
