# Supplementary material for: Identification of SPRYD4 as a tumour suppressor predicts prognosis and correlates with immune infiltration in cholangiocarcinoma
Source: BMC Cancer. 2023 May 4;23:404. doi: 10.1186/s12885-023-10810-9 (PMC10161465; doi:10.1186/s12885-023-10810-9)
Supplement: Supplementary file 1 — Additional file 1: Table S1. Information on PCR primer oligonucleotide sequences. [file 12885_2023_10810_MOESM1_ESM.docx]

Table S1. Information on PCR primer oligonucleotide sequences

|  |  |
| --- | --- |
| **ID** | **Sequence (5’- 3’)** |
| SPRYD4 F | TGCACGTTCTTTGCGCTTG |
| SPRYD4 R | GTTTGAAACTGACGCCTCTCT |
| CDK2 F | CCAGGAGTTACTTCTATGCCTGA |
| CDK2 R | TTCATCCAGGGGAGGTACAAC |
| CCNA2 F | CGCTGGCGGTACTGAAGTC |
| CCNA2 R | GAGGAACGGTGACATGCTCAT |
| BCL2 F | GGTGGGGTCATGTGTGTGG |
| BCL2 R | CGGTTCAGGTACTCAGTCATCC |
| BIRC5 F | AGGACCACCGCATCTCTACAT |
| BIRC5 R | AAGTCTGGCTCGTTCTCAGTG |
| GAPDH F | GGAGCGAGATCCCTCCAAAAT |
| GAPDH R | GGCTGTTGTCATACTTCTCATGG |
