# Supplementary material for: Identification of SPRYD4 as a tumour suppressor predicts prognosis and correlates with immune infiltration in cholangiocarcinoma
Source: BMC Cancer. 2023 May 4;23:404. doi: 10.1186/s12885-023-10810-9 (PMC10161465; doi:10.1186/s12885-023-10810-9)

**Supplementary Figures**

**Figure S1. SPRYD4 expressions in pan-cancer.** The analyses of SPRYD4 expression in several cancer types in GEPIA database.


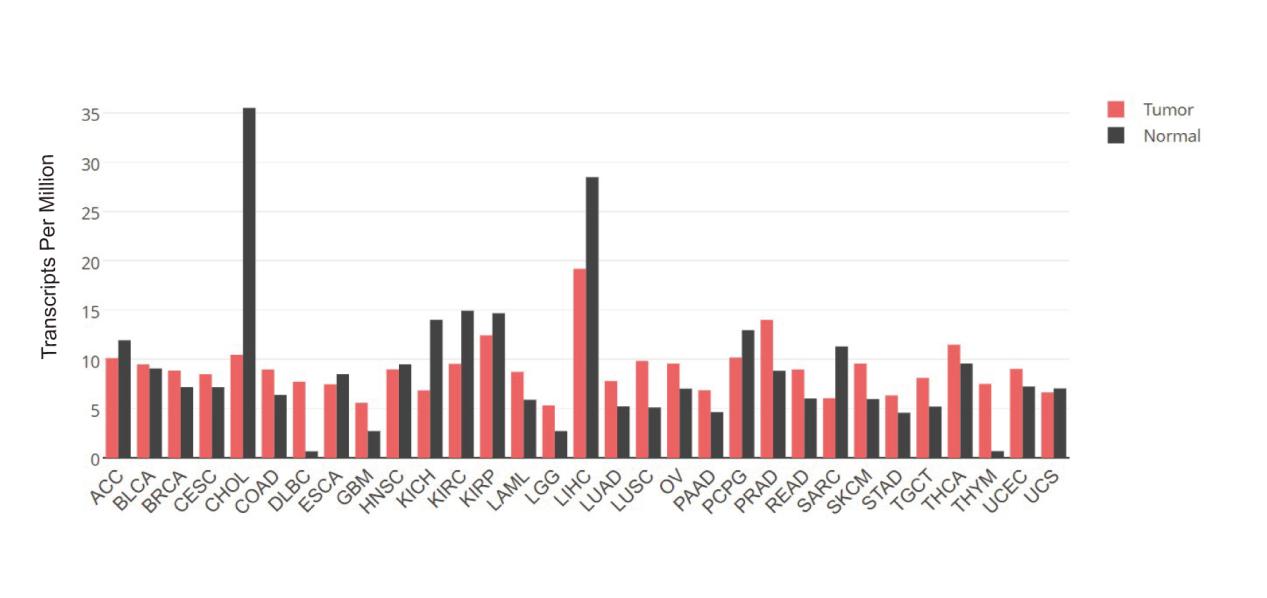


**Figure S2. Survival analyses of SPRYD4 in other cancer types.** Kaplan-Meier analyses showed patients with low SPRYD4 expression had inferior overall survival in kidney renal clear cell carcinoma (KIRC), kidney renal papillary cell carcinoma (KIRC), liver hepatocellular carcinoma (LIHC), pancreatic adenocarcinoma (PAAD) and lung squamous cell carcinoma (LUSC).


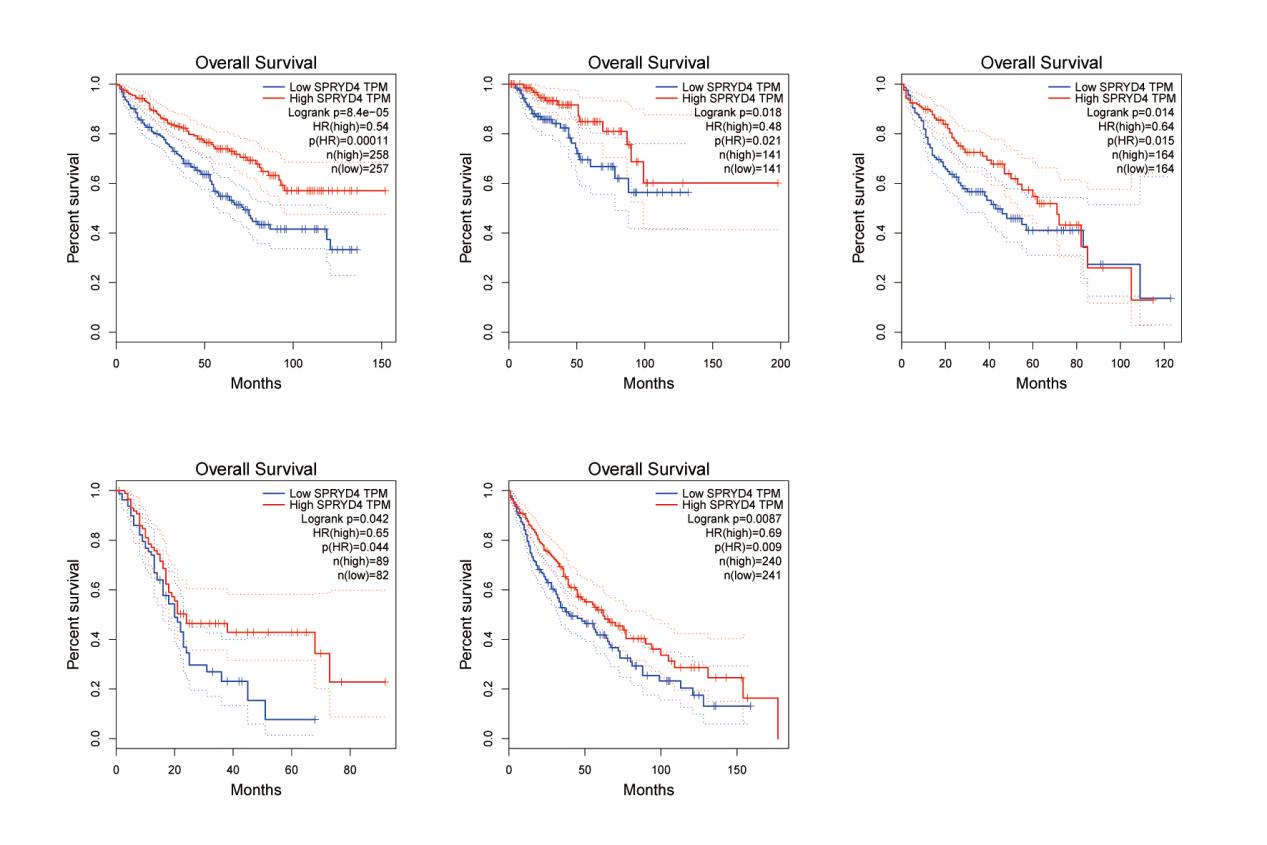


**Figure S3. Correlations of SPRYD4 with immunomodulators in ACC.** (A) Correlations between immunostimulators and SPRYD4 expression shown by TISIDB database. (B) Correlations between immunoinhibitors and SPRYD4 expression shown by TISIDB database. (C) Top 3 immunostimulators with greatest negative Spearman’s correlation with SPRYD4. (D) Top 3 immunoinhibitors with greatest negative Spearman’s correlation with SPRYD4.


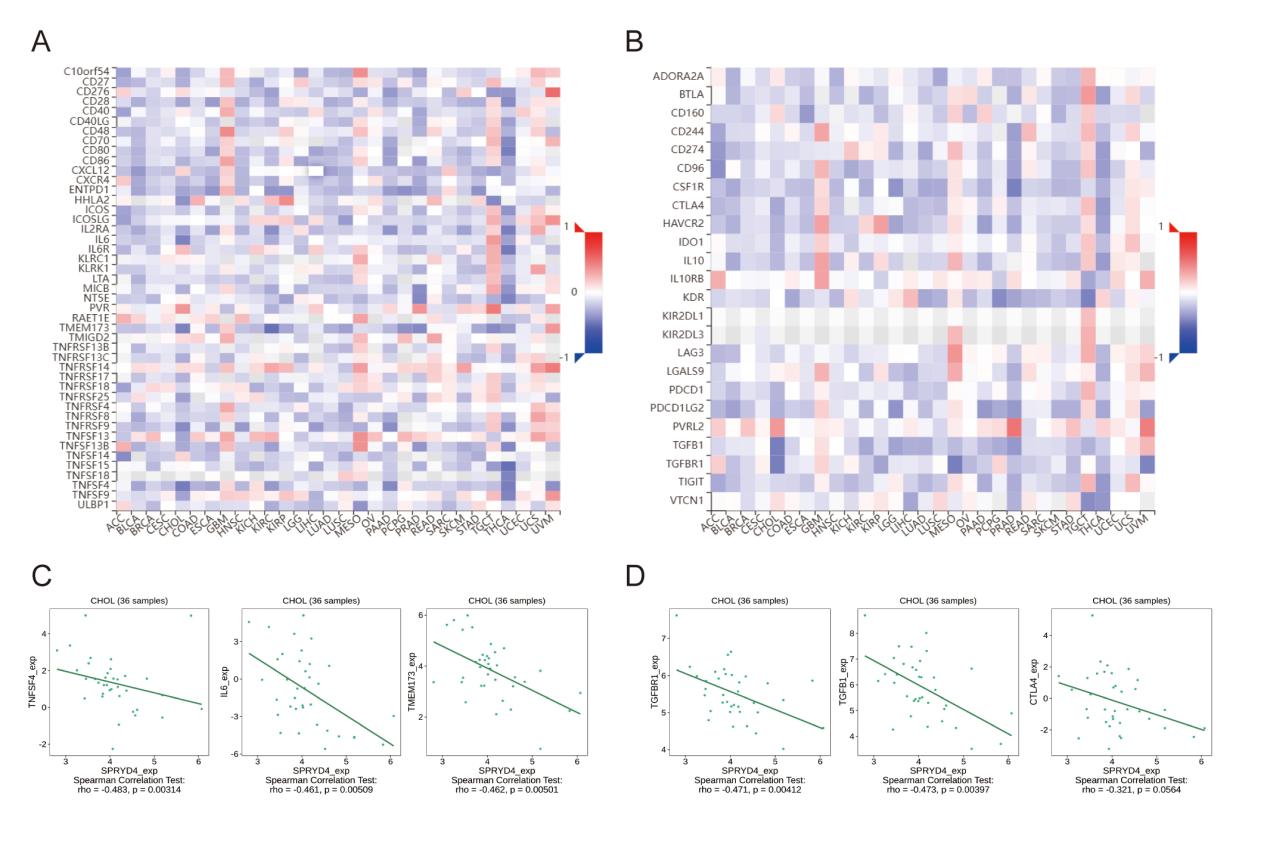

Supplement: Supplementary file 2 — Additional file 2: Figure S1. SPRYD4 expressions in pan-cancer. The analyses of SPRYD4 expression in several cancer types in GEPIA database. Figure S2. Survival analyses of SPRYD4 in other cancer types. Kaplan-Meier analyses showed patients with low SPRYD4 expression had inferior overall survival in kidney renal clear cell carcinoma (KIRC), kidney renal papillary cell carcinoma (KIRC), liver hepatocellular carcinoma (LIHC), pancreatic adenocarcinoma (PAAD) and lung squamous cell carcinoma (LUSC). Figure S3. Correlations of SPRYD4 with immunomodulators in ACC. (A) Correlations between immunostimulators and SPRYD4 expression shown by TISIDB database. (B) Correlations between immunoinhibitors and SPRYD4 expression shown by TISIDB database. (C) Top 3 immunostimulators with greatest negative Spearman’s correlation with SPRYD4. (D) Top 3 immunoinhibitors with greatest negative Spearman’s correlation with SPRYD4. [file 12885_2023_10810_MOESM2_ESM.docx]
